# Umbrella Sampling Workflows for Fast-Converging PMF Calculations without Artificial WHAM Constraints

**DOI:** 10.1021/acs.jctc.6c00246

**Published:** 2026-05-06

**Authors:** Bjarne Feddersen, Philip C. Biggin

**Affiliations:** Structural Bioinformatics and Computational Biochemistry, Department of Biochemistry, 6396 University of Oxford, South Parks Road, Oxford OX1 3QU, U.K.

## Abstract

Umbrella sampling is a powerful tool to investigate the underlying free-energy landscapes that govern the permeation properties of small molecules through complex lipid bilayers. However, obtaining converged potentials of mean force (PMFs) remains an issue due to sampling limitations, and as a result, enhanced sampling approaches are constantly being developed. Here, we benchmark umbrella sampling workflows and test different options for window generation, sampling, and statistical estimation of PMFs against each other to arrive at recommendations to improve PMF convergence speeds and minimize required computational resources. We also report large errors introduced into PMFs by enforcing their symmetry and/or periodicity when sampling is insufficient, leading us to caution against the use of these constraints in future works.

## Graphical abstract

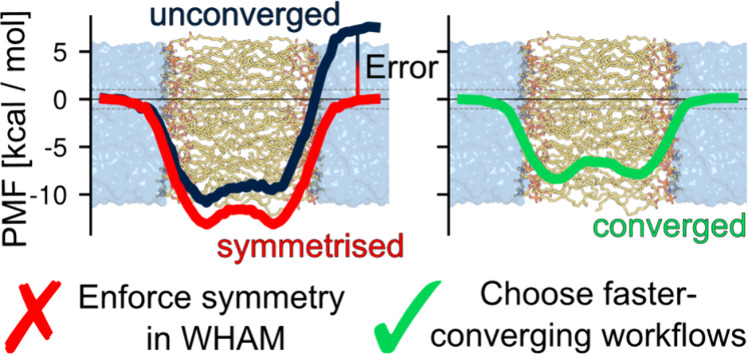

## Introduction

Most drugs have to pass cell membranes on the way to their site of action.^1–3^ Hydrophobicity and membrane partitioning behavior strongly influence a drug’s absorption, distribution, metabolism, and excretion properties (ADME) and need to be considered early on in drug development.^4,5^ The importance of these factors was distilled into Lipinski’s ″rule of 5″,^6^ which offers criteria to predict oral bioavailability based on a compound’s size, number of hydrogen donor and acceptor groups, and octanol–water partition coefficient (logP). logP values are useful predictors and can readily be calculated from chemical structures. However, they are often insufficient determinants of membrane permeation.^7,8^ Membranes are highly complex assemblies whose precise composition can vary between species and cell types.^9^ Lipids affect overall membrane behavior through their different head groups, fatty acid chain lengths, degrees of saturation, and rigidity. Furthermore, integral membrane proteins add additional complexity. It is thus no surprise that the correlation between octanol–water partition coefficients and membrane permeation is often limited. This is sometimes attributed to physical effects of the membrane composition compared to a pure octanol phase^7,10–12^ and other times linked to transporter protein-mediated uptake.^13,14^

Molecular dynamics simulations can be used to more accurately study the effects of membrane composition on solute partitioning,^15–17^ but slow convergence of free energy calculations has been and continues to be an issue. It has thus been common practice to enforce symmetry or periodicity of the potential of mean force (PMF) as a boundary condition. However, there are undoubtedly errors that arise from these constraints.^18,19^ Though these errors can be minor,^19^ their magnitude is hard to determine. They are thus difficult to account for, meaning that the use of these constraints should ideally be avoided.

The general umbrella sampling workflow consists of window generation, data collection, and PMF calculation, with a variety of tools available for each of these three steps. The influence of different window generation methods on the PMF of dissociation processes was recently described by You et al. for example.^20^ Here, we report the influence of the choice of tools on the convergence speed of PMFs by comparing two different tool options for each step. We were particularly interested in the reduction of hysteresis and acceleration of PMF convergence. For that purpose, we tested nonstandard window generation against window seeding from steered MD, Simulated Tempering-enhanced Umbrella Sampling (STeUS)^21^ against standard umbrella sampling, and PMF calculation with MBAR employing temperature reweighting against WHAM without it. Each component’s effectiveness was determined by calculating the PMFs of permeation of eight small molecules (Figure 1) through two different lipid bilayers (Table 1). A focus lay on obtaining symmetrical and periodic PMFs without the need to enforce these characteristics as boundary conditions.

**Figure 1:**
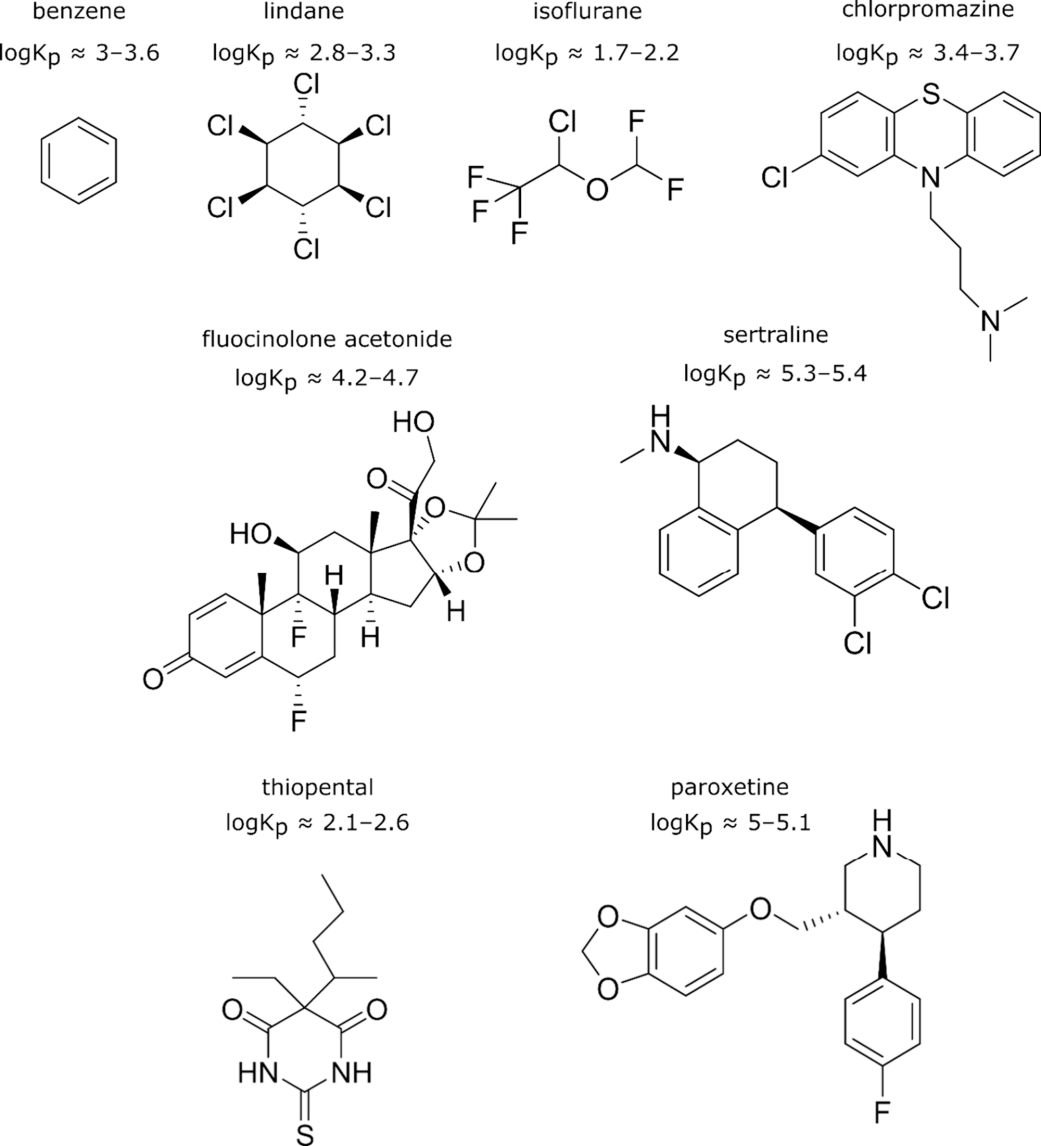
Ligands used in this study. They represent a broad section of chemical space and have experimental evidence that cholesterol content in membranes reduces membrane partitioning. Approximate partition coefficients (log*K*_p_) for different membrane contexts are listed. Further details on these coefficients and their references are given in Table S1.

**Table 1:** System Composition

	pure POPC	POPC + 30 mol % cholesterol
# POPC	48	42
# cholesterol		18
# Na^+^	7	8
# Cl^–^	7	8
# H_2_O	2916	3270
# atoms, total	15,194	16,786
box size [nm]	4 × 4 × 10	4.25 × 4.25 × 10

Permeation through two different lipid bilayers was tested in this study: a pure 1-palmitoyl-2-oleoyl-*sn*-glycero-3-phosphocholine (POPC) bilayer and a cholesterol-doped POPC bilayer with 30 mol % cholesterol. Phosphatidylcholine lipids are the most common class of phospholipids in mammalian cell membranes.^22^ POPC is a useful model lipid because it combines a *cis*-unsaturated (oleic acid, 18:1) and a saturated (palmitic acid, 16:0) fatty acid, giving it intermediate behavior in terms of membrane rigidity and fluidity compared with fully saturated and poly unsaturated lipids.^23^ The sterol lipid cholesterol constitutes 20 to 50 mol % of mammalian membranes.^24–26^ As such a significant component, it strongly affects overall membrane behavior. Through intercalation between phospholipids, it enforces increased lipid tail order, which in turn increases the membrane thickness and reduces the area per lipid. Both of these effects increase the barrier of permeation of solutes.^24,27^ This increased barrier of permeation was previously demonstrated as reduced partitioning into bilayers containing cholesterol for all eight compounds tested in this study: benzene,^28^ lindane,^29^ isoflurane,^30^ thiopental,^31^ fluocinolone acetonide,^8^ chlorpromazine,^32^ paroxetine,^33^ and sertraline^33^ (Figure 1 and Table S1). These compounds were chosen as they cover a broad range of chemical complexity and functional groups.

## Methods

### Simulation Details

The two membrane systems (Table 1 and Figure 2) were prepared with CHARMM-GUI,^34–38^ choosing the SLipids parameters^39,40^ for POPC and cholesterol and TIP3P^41^ as the water model. Small molecules were parametrized with GAFF2.^42^ While partitioning into lipid environments has been reported for all ligands in this study, some of them can exist as charged species at physiological pH. A possible model for membrane permeation of such compounds relies on the fact that pK_a_ values depend on the chemical environment, and membrane proximity can shift the protonation equilibrium sufficiently to allow uncharged species to form and partition into the hydrophobic membrane.^43^ As the focus of this study lies on the calculation of converged PMF profiles rather than comparison to experimental data, the GAFF2 parameters of uncharged ligand species were used without further modification, regardless of each compound’s p*K*_a_ value.

**Figure 2:**
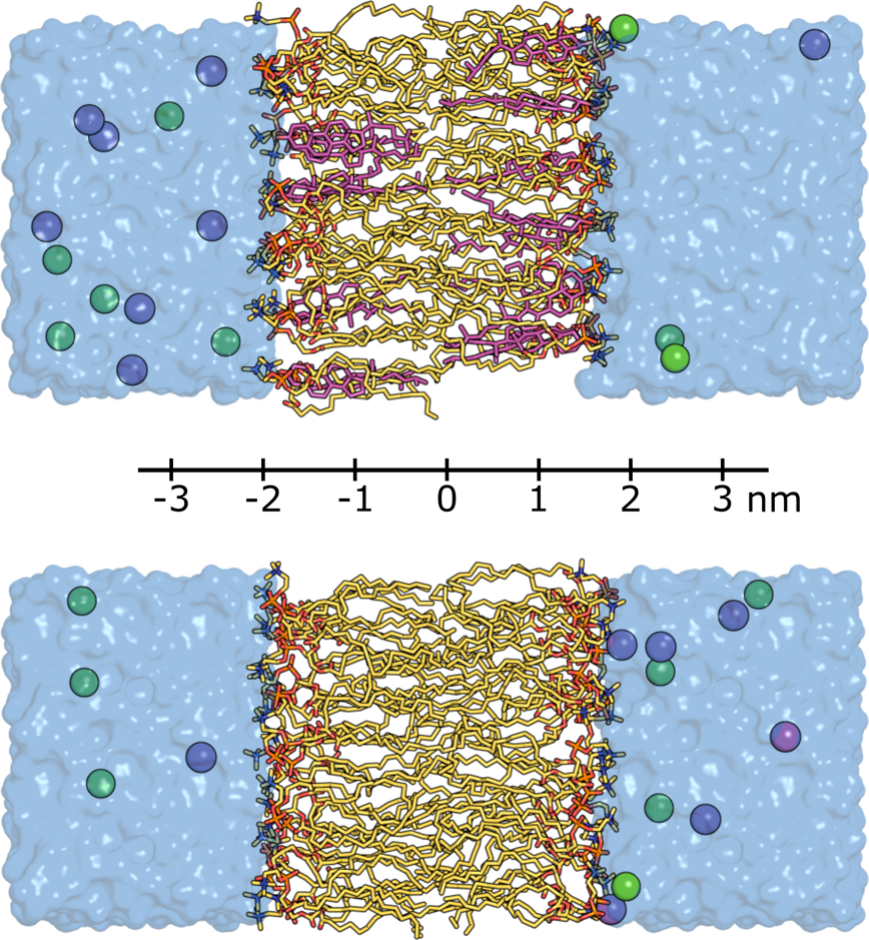
Cholesterol-doped POPC bilayer (top) and pure POPC bilayer (bottom) systems used in this study. System composition is shown in Table 1. Cholesterol and POPC are shown in stick representation (magenta and yellow, respectively), water is shown as a blue volume, and sodium and chloride ions are shown as purple and green spheres, respectively. The reaction coordinate used in the umbrella sampling simulations is defined as the center-of-mass distance between ligand and membrane and is illustrated here.

Simulations were carried out with GROMACS 2021.7,^44–49^ using a time step of 2 fs. The LINCS algorithm^50^ was used to constrain bonds to hydrogen, except in the case of water, which was constrained with SETTLE.^51^ Long-ranged electrostatic interactions were calculated with the smooth particle mesh Ewald approach,^52^ while a Verlet cutoff scheme was applied to short-ranged interactions. A pressure of 1 bar was maintained in all simulations using a semi-isotropic C-rescale barostat^53^ with a coupling constant of 5 ps. Temperature was maintained at values in a range from 310 to 358 K with the V-rescale thermostat,^54^ as indicated later, using a 1 ps coupling constant.

Both bilayers were equilibrated in a stepwise procedure that gradually lowered the position and dihedral restraints on the lipids (Table 2). In a final equilibration step, the systems were simulated for 500 ns without any restraints to ensure proper equilibration and phase behavior of the lipid bilayer.

**Table 2:** Details of the Lipid Bilayer Equilibration Procedure

			restraint force constants	
equilibration step	number of simulation steps	timestep [fs]	head groups	POPC tail dihedrals
1	125,000	1	1000	1000
2	125,000	1	400	400
3	125,000	1	400	200
4	250,000	2	200	200
5	250,000	2	40	100
6	250,000,000	2	0	0

The reaction coordinate used in umbrella sampling was the center-of-mass distance between small molecule and membrane in the membrane-normal direction. Harmonic umbrella potentials of 1000 kJ mol^–1^ nm^–2^ were applied to restrain the ligand to discrete values along this coordinate. We observed the movement of cholesterol between the two membrane leaflets, particularly at the higher temperatures sampled in this study (Figure S1). To prevent cholesterol flip-flop, harmonic flat-bottom restraints were applied to the oxygen atom of the cholesterol headgroup. These restraints applied a force of 1000 kJ mol^–1^ nm^–2^ if the headgroup moved more than 1.3 nm from its starting position along the membrane normal, ensuring free and unrestrained movement of cholesterol within its starting leaflet but preventing flip-flopping that would complicate PMF calculations.

### Umbrella Window Generation

Seventy-six individual umbrella windows along the reaction coordinate were generated for each ligand and bilayer combination. The effectiveness of two different approaches, starting from the equilibrated bilayers, is compared in this study.

#### Alchemical Growth of Ligands along the Reaction Coordinate

One common problem in umbrella sampling simulations is hysteresis,^55^ which is the dependence of an estimated PMF or free-energy profile on the path used to generate the sampling windows or on the prior history of the system. Because the equilibrium free energy is a state function, such a history dependence indicates a convergence error, often caused by insufficient sampling or slow hidden degrees of freedom. Overcoming hysteresis artifacts is therefore often an important step in obtaining reliable free-energy profiles. The window generation method described here aims to reduce hysteresis artifacts by avoiding the steered MD step commonly used in window generation. In a procedure inspired by the Alchembed method for embedding proteins into lipid bilayers,^56^ the ligand coordinates are programmatically manipulated to position the small molecule at desired reaction coordinate values along the membrane-normal. The resulting clashes are resolved by gradually switching on the Lennard-Jones (LJ) interactions of the ligand. We used the soft-core LJ implementation in GROMACS with α = 0.1 and Δλ = 0.001 to fully switch on LJ interactions in 1000 simulation steps, during which harmonic restraints are placed on the ligand to maintain the desired reaction coordinate value.

#### Steered-MD-Based Umbrella Windows

More commonly, the initial system conformations for umbrella sampling are obtained from a steered MD (sMD) trajectory. In this study, the small molecules were steered through the membrane bilayer using the GROMACS pull code with a harmonic potential of 1000 kJ mol^–1^ nm^–2^ and a pull rate of 0.1 nm/ns. Pulling simulations started with the ligand roughly 3.75 nm above the bilayer’s center of mass. MDAnalysis^57,58^ was then used to extract trajectory frames with equal spacing along the reaction coordinate.

### Umbrella Sampling Simulation Methods

Thus, two sets of umbrella windows were generated for each combination of the ligand and lipid bilayer. Regardless of the simulation method chosen, each umbrella window simulation was run for 100 ns, unless otherwise stated.

#### Standard Umbrella Sampling

Standard umbrella sampling simulations were carried out at 310 K with the parameters described above. The leapfrog integrator was used.

#### Simulated Tempering-Enhanced Umbrella Sampling

One common problem in umbrella sampling simulations is that the degrees of freedom of the system outside of those that are biased with the umbrella potential can also significantly affect sampling and thus PMF convergence. If these orthogonal degrees of freedom are slow to sample, then PMF convergence is negatively affected. Simulated Tempering-enhanced Umbrella Sampling (STeUS)^21^ is a method designed to enhance sampling of such slow orthogonal degrees of freedom. It makes use of the simulated tempering technique, in which moves along a predefined temperature ladder are periodically attempted and then accepted or rejected based on a Metropolis criterion. Simulating at higher temperatures allows energy barriers to be overcome more quickly, which improves sampling both along the reaction coordinate and along the orthogonal degrees of freedom that often hinder convergence.^59^ However, as the PMF is temperature dependent, only data collected at the temperature of interest can be used to calculate the PMF of interest, and the trajectory has to be postprocessed to ensure only ground-state information is used in its calculation. This complicates the use of simulated tempering for umbrella sampling: The wider the chosen temperature ladder is, the more time spent at higher temperatures to allow the overcoming of energy barriers that slow convergence. At the same time, however, less simulation data are available at the temperature of interest. In recognition of this trade-off, the STeUS method implements a modified Metropolis criterion, in which the user can specify the desired occupancy of the lowest temperature state. Its original authors report that for ligands in which the main hindrance of convergence is likely to be orthogonal energy barriers, this ground-state occupancy should be set relatively low to grant sufficient time at higher temperatures. Ligands that are fast to converge, on the other hand, should be sampled with a higher ground-state occupancy instead.

In this study, two sets of STeUS simulations were run for each combination of ligand, membrane, and window generation method. To account for different ligand behavior, ground-state occupancies of 20 and 40% were tested. Initial weights were estimated from brief simulations at each temperature, as described by Park and Pande.^60^ Moves along the temperature ladder were attempted every 500 steps, and the STeUS-modified Wang–Landau algorithm was employed to update the weights of each temperature state. The velocity–Verlet integrator was used for the integration of the equations of motion. The temperature ladder bridged 310 and 358 K in 6 K increments, with 20 or 40% of the simulation time spent at 310 K.

### Calculation of Potentials of Mean Force

For the calculation of the PMF, the first 5 ns of each umbrella window was discarded for equilibration unless otherwise stated.

#### WHAM

GROMACS’s own WHAM implementation—gmx wham^18^—was used here to calculate the PMF based on the runs’ TPR input files and the reaction coordinate output files. In the case of STeUS simulations, the output files were preprocessed, and only the subset of data collected at the temperature of interest was used in WHAM.

During the calculation of the PMF, gmx wham can enforce the symmetry and periodicity of the profile. For a perfectly symmetrical, converged PMF, these mathematical constraints would not alter the profile’s shape. In the case of insufficient sampling, however, unconverged profiles must be altered significantly to satisfy these boundary conditions. To quantify the errors that result from the enforcement of symmetry and periodicity of the PMF, a set of PMF calculations was carried out with the -sym,-cycl, and -ac flags passed to the gmx wham. To see the effects on nonconverged profiles, these PMF calculations were based on 10 ns instead of 100 ns per window. Standard umbrella sampling was run on sMD-based windows, and the first 200 ps was discarded for equilibration. This was repeated in triplicate with three independent sMD trajectories.

#### MBAR

In the original STeUS publication, data collected at higher temperatures was discarded outright. With the ground-state occupancies tested in this study, this means that 60–80% of the simulation time of each window would not be used. However, in the case of small enough simulation systems, statistical reweighting can be used to inform the PMF at the ground-state temperature.^61,62^ Reweighting between different conditions is carried out via the reduced potential energy *u*(*x*) of a configuration of system *x*. In the case of umbrella sampling in the NPT ensemble, the system’s temperature *T* (in form of the inverse temperature β = 1/*kT*), pressure *p*, volume *V*, and umbrella bias potential *B* have to be combined with the potential energy *U* to calculate the reduced potential energy u as

u(x)=β(U(x)+pV(x)+B(x))

In the application to STeUS simulations, there are (number of temperatures) × (number of umbrella windows) states that have to be considered in MBAR. The reduced potential energy of each uncorrelated configuration *x* in the trajectory of each state is used to calculate the potential of mean force of the unbiased process at the ground-state temperature. This depends on sufficient overlap between the state from which a configuration is sampled and the state to which reweighting is desired. In the case of the umbrella biases *B*, it is easy to see that adjacent umbrella windows will contain information about each other that can be extracted in reweighting but that windows that are separated further will contain less information about each other. This is ultimately caused by large differences in the reduced potential energy between the two states, which result in a near-zero weight in the reweighting procedure. The formulation of the reduced potential energy shows that reweighting between different temperatures is possible in principle as well. Here, we set out to determine if such temperature reweighting is useful in practice or if the differences in reduced potential energies are too large to allow meaningful contributions to the calculation of the PMF at the ground state.

To evaluate the effectiveness of temperature reweighting to the STeUS simulations carried out here, the pymbar implementation of MBAR^63^ was used to calculate PMFs at the ground-state temperature using all available simulation data. Further information on the effectiveness of temperature reweighting is included in Figure S2.

### Umbrella Sampling Workflows

In general, the umbrella sampling workflow can be broken down into three components. The individual configurations of the system have to be generated, the (biased) simulation data have to be collected from each configuration, and the underlying, unbiased potential of mean force has to be calculated through statistical reweighting. In this study, umbrella sampling workflows are assembled from combinations of two different options for each of these three workflow components (Figure 3), as described above. In particular, windows generated from steered MD trajectories are compared with alchemically grown configurations. A standard umbrella sampling protocol is compared with Simulated Tempering-enhanced Umbrella Sampling (STeUS) with different settings. Moreover, the effectiveness of temperature reweighting of STeUS generated trajectories with MBAR is quantified against the calculation of PMFs from data collected at the temperature ground state only, which were carried out with WHAM.

**Figure 3:**
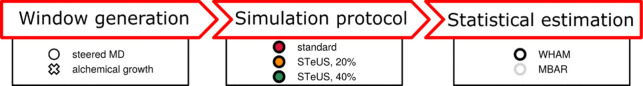
The three steps of an umbrella sampling study, and the individual components compared in this work. The nomenclature defined here is used consistently throughout Figures 4, 5, 6, 7, and 8. Window generation methods are distinguished through shape, simulation protocols through fill color, and the use of temperature reweighting (MBAR) or ground-state information only (WHAM) with the outline color.

Many other enhanced sampling approaches to accelerate convergence have been developed over the years. Various replica exchange methods, for example, have been reported to drastically improve convergence. Applied to umbrella sampling, a complete set of umbrella windows is still required to sample the reaction coordinate. However, during the simulation, the windows are not independent. Instead, they are run in parallel, and exchanges of the Hamiltonian of neighboring windows—including the umbrella bias potential—are attempted periodically. These exchanges have been shown to improve convergence dramatically.^64^ This approach can be extended with replica exchange moves along a temperature ladder, as well as along the umbrella windows. Such two-dimensional replica exchange methods have, for example, been used to enhance sampling efficiency in the study of RNA folding.^65^ However, temperature replica exchange suffers from the same underlying problem as temperature reweighting, as described above: The exchange probability between temperatures is strongly reduced in larger systems, requiring more replicas at narrower spacing. In an effort to circumvent this issue, replicas in the Replica Exchange with Solute Tempering (REST) approach differ in their Hamiltonians, with interaction potentials scaled to simulate the effects of higher temperature without affecting the potential energy of the system as strongly.^66,67^ This approach reduces the number of replicas required to span a comparable effective temperature range.

While all three of these replica exchange methods have been demonstrated to improve convergence of free-energy calculations, we have chosen to focus on STeUS in this study because of a number of inherent advantages of the method. Unlike the parallel replica exchange methods, each STeUS umbrella window is fully independent. Since no exchanges occur between windows, no communication between windows is required at runtime. This reduces hardware constraints on the number of windows and temperatures that can be tested at the same time and allows windows to be simulated across different computational resources. It is also straightforward to extend simulations in individual windows or add new windows, as required.

### Convergence Criteria

Commonly, convergence of the potential of mean force profiles is assessed by monitoring how the profile changes as additional simulation data are included in the analysis. When the PMF no longer changes appreciably upon inclusion of more data, it is often regarded as converged. However, this criterion alone does not guarantee that the PMF is physically correct since effects such as hysteresis or insufficient sampling of slow orthogonal degrees of freedom can yield a profile that appears numerically stable while remaining physically inaccurate. Therefore, in this study, PMF convergence was assessed using two criteria, taking the nature of the simulation system into account. As the simulation systems are symmetric, the PMF profiles should be symmetric about the center of the system at a reaction coordinate value ξ = 0 nm, as well. We quantified this symmetry through the mean absolute error of symmetry, MAE_sym_, as

$$\mathrm{MA}E_{\mathrm{sym}}=\frac{1}{N}\overset{\xi_{\max}}{\underset{\xi =0}{\sum }}\left|W\left(\xi \right)-W\left(-\xi \right)\right|$$

where *N* is the total number of reaction coordinate pairs [ξ, – ξ], *W*(ξ) is the PMF, and ξ_max_ is the largest value of the reaction coordinate that was sampled.

The second error considered here is the difference in the PMF between the two bulk water components. The PMF at the minimum reaction coordinate value W­(ξ_min_) is defined as 0 kcal mol^–1^. As periodic boundary conditions are used in these simulations, the bulk water regions on either side of the membrane are identical and the PMF should have the same value at ξ_min_ and ξ_max_. The sum of all sampling errors can thus be estimated in the offset of *W*(ξ_max_) from 0 kcal mol^–1^. Since this bulk offset can give immediate clues about the degree of correctness at a glance, it is chosen as the second convergence criterion in this study, even though it is already captured by MAE_sym_. We considered a PMF to be converged if both error criteria measured less than 1 kcal mol^–1^, a commonly cited value for chemical accuracy.^64^ In this way, our definition of convergence goes beyond numerical consistency and takes the physical nature of the system into account, providing a more accurate depiction of its energetics. The convergence speed of each system was then determined from the amount of simulation time needed to reach convergence (Figure 4). The effectiveness of the different combinations of tools in the umbrella sampling workflow is then compared by this value. This abstract measure of convergence speed allows the performance of different umbrella sampling workflows to be readily compared as all factors influencing convergence are distilled into a single data point. The less time a workflow needed for both errors to fall below the cutoff of chemical accuracy, the better.

**Figure 4:**
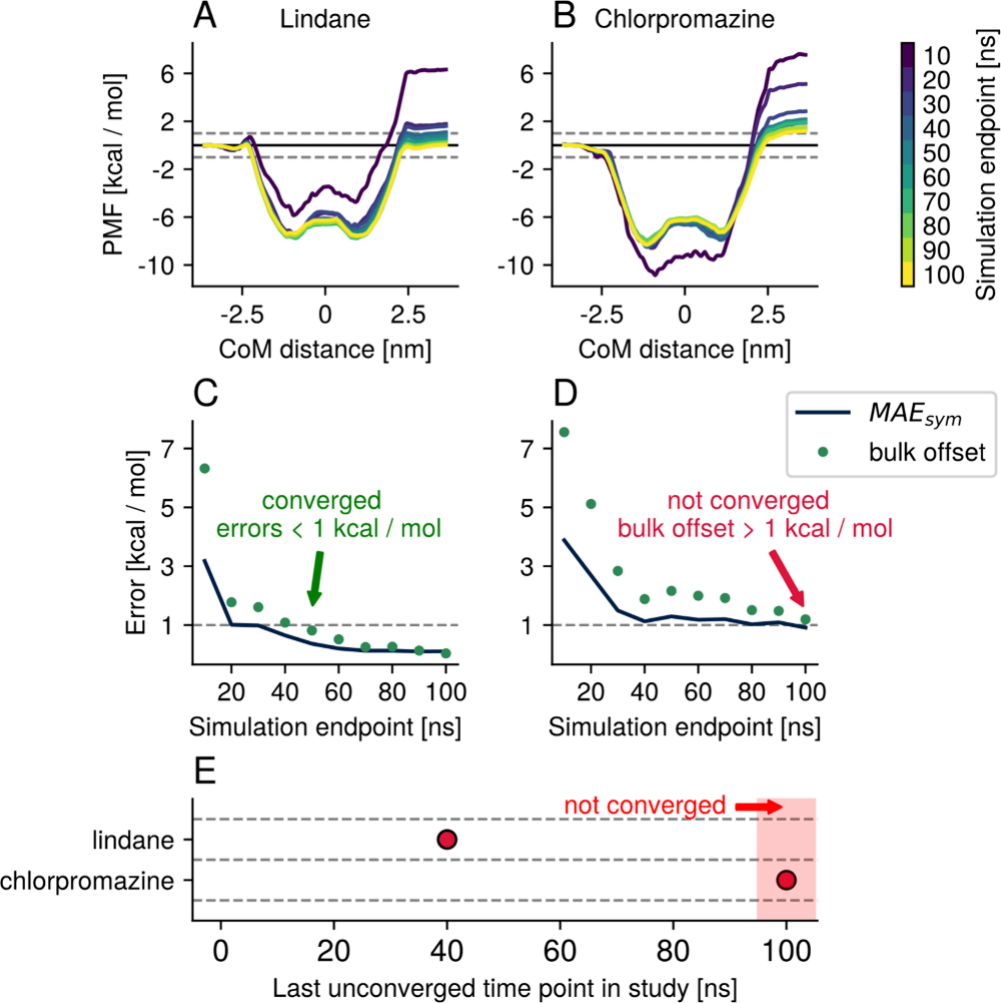
Illustration of the determination of convergence speed. The PMFs for lindane (A) and chlorpromazine (B) are calculated for subsets of the available data. The first 5 ns of each window is discarded for equilibration, and the calculations are carried out over data up to the end point indicated in the color bar. The mean absolute error of symmetry (MAE_sym_) and the bulk offset error are calculated, and the PMF is considered converged when both are below 1 kcal mol^–1^ (C, D), as this is a common measure for chemical accuracy. Improvements below these thresholds of chemical accuracy are not quantified in this study. The convergence speed is then described through the simulation time per window required for both error measures to converge below the chemical accuracy. This error analysis is carried out for all PMFs calculated in this study, and convergence speeds are used to compare across the conditions. In this example, the PMF for lindane converged according to our metrics of chemical accuracy within 50 ns. The PMF for chlorpromazine, on the other hand, may appear numerically converged in (B) and (D), but as one of the error metrics we defined to ensure the physical correctness of the results is beyond chemical accuracy, we consider this PMF not to be converged. Convergence speeds are compared throughout this study by a single data point: the last time point at which the PMF was not converged (E). Using this metric, conditions that do not lead to convergence are included at 100 ns, and the smaller the time point, the faster the PMF of a tested condition converged within the chemical accuracy. The marker for a PMF that converges within 10 ns of simulation time would be placed at 0 ns in these plots, as the first time point tested had already converged. This convergence speed analysis was carried out for all workflow combinations tested in this study, using the iconography shown in Figure 3.

### Calculation of Partition Coefficients

Partition coefficients were calculated from the PMF profiles obtained in this study. These coefficients are not necessarily expected to match experimental results: Since the aim of this study is the improvement of convergence speeds rather than the correctness of PMFs, no optimization of ligand parameters was carried out. Instead, they are calculated here to facilitate comparisons between results collected in the cholesterol-free and cholesterol-doped POPC bilayers. The free energy of partitioning Δ*G*_part_ into the region of the reaction coordinate ξ between ξ_low_ and ξ_high_ can be obtained from the PMF via integration^21^ as

$$\Delta G_{\mathrm{part}}=-\mathrm{\beta ln}\left(\frac{1}{\xi_{\mathrm{high}}-\xi_{\mathrm{low}}}\int_{\xi_{\mathrm{low}}}^{\xi_{\mathrm{high}}}e^{-\beta W(\xi )}d\xi \right)$$

where $\beta =\frac{1}{k_{B}T}$ . ξ_low_ and ξ_high_ were set to −1.5 and 1.5 nm, respectively, which roughly correspond to the hydrophobic core of the membrane. The integration of the PMF *W*(ξ) was carried out with the trapezoidal rule. From this free energy, the partition coefficient *K*_p_ can be calculated as

$$K_{p}=e^{-\beta \Delta G_{\mathrm{part}}}$$

### Computational Cost

The reaction coordinates in both bilayer systems were spun by 76 umbrella windows. Ligand spacing between adjacent windows varied by 0.1 nm. Table 3 lists the total simulation time required for a PMF for each simulation end point. As an example, the PMF for lindane shown in Figure 4 required 50 ns of simulation time per window to converge according to our error metrics. This is equivalent to a total simulation time of 3.8 μs.

**Table 3:** Total Simulation Time Needed for PMF Convergence Is Calculated from the Simulation Time of Each Window

simulation time per window [ns]	total simulation time for PMF [μs]
10	0.76
20	1.52
30	2.28
40	3.04
50	3.8
60	4.56
70	5.32
80	6.08
90	6.84
100	7.6

## Results

### Alchemically Grown Initial Configurations Outperform sMD-Based Windows

To assess the impact of the window generation method on PMF convergence, the amount of simulation time of the standard umbrella sampling protocol required to reach convergence for each compound and membrane was determined for both sets of windows (Figure 5). In this comparison, alchemically grown umbrella windows lead to convergence as fast as or faster than sMD-based windows in 13 out of the 16 compound-bilayer combinations. The effect on convergence speeds differs between conditions. While the differences for benzene and sertraline amount to at most 10 ns of simulation time, much larger improvements in convergence speed can be seen for the other compounds, particularly for paroxetine. While alchemically grown umbrella windows do not universally improve convergence speeds, they do appear to outperform sMD-based windows more often than not. Strikingly, there are compound-bilayer combinations in which umbrella sampling on sMD-based windows does not lead to converged PMFs within the simulation time tested here. These combinations—fluocinolone in both bilayers, chlorpromazine in the pure POPC bilayer, and paroxetine in the cholesterol-doped bilayer—can however, be made to yield converged PMFs through the use of alchemically grown windows, instead.

**Figure 5:**
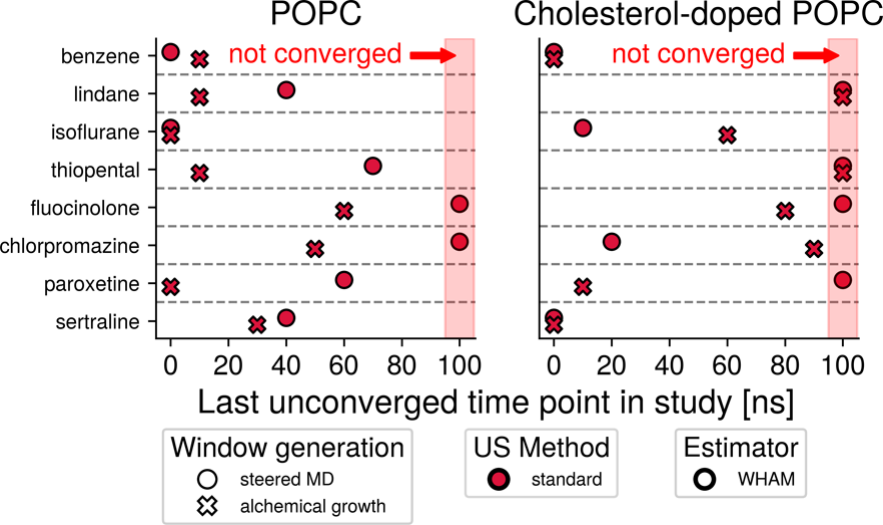
Influence of varying the window generation method in isolation. The convergence speed analysis described in Figure 4 was carried out to determine the influence the two window generation methods tested in this study have on convergence speed on their own. Windows seeded from sMD (circles) or alchemical growth (crosses) were simulated with standard umbrella sampling (red color), and the PMFs were calculated with WHAM (black outline). This comparison showed that alchemical growth of initial configurations performs as well as or better than sMD-based window seeding in 13 out of the 16 combinations of bilayer and small molecule tested in this study. Markers placed at 100 ns represent umbrella sampling workflows that did not lead to convergence.

### Independent Confirmation that STeUS Improves Convergence

In its original publication, STeUS was reported to outperform standard umbrella sampling in all tested conditions.^21^ Here, we find that STeUS can indeed be a powerful accelerant of PMF convergence. As originally reported, the choice of ground-state occupancy is an important parameter with a large impact on convergence speeds. We tested ground-state occupancy values of 20 and 40% and found that STeUS simulations performed on sMD-based windows lead to as fast or faster convergence than standard simulation parameters in 13 out of the 16 compound-bilayer combinations in a strongly ground-state occupancy-dependent manner (Figure 6). Dramatic improvements can be observed, for example, for fluocinolone and chlorpromazine in the pure POPC bilayer and for paroxetine in the cholesterol-doped POPC bilayer, while benzene and isoflurane show mostly invariant behavior. The importance of the choice of ground-state occupancy is nicely illustrated by thiopental in the pure POPC bilayer. Standard umbrella sampling leads to PMF convergence after 70 ns of simulation time. STeUS with 40% ground-state occupancy reduces the required simulation time, and convergence is reached after 30 ns, instead, while a ground-state occupancy of 20% prevents convergence within 100 ns. Our results constitute an independent verification of the power of Sousa et al.’s STeUS method and further emphasize the importance of the choice of ground-state occupancy.

**Figure 6:**
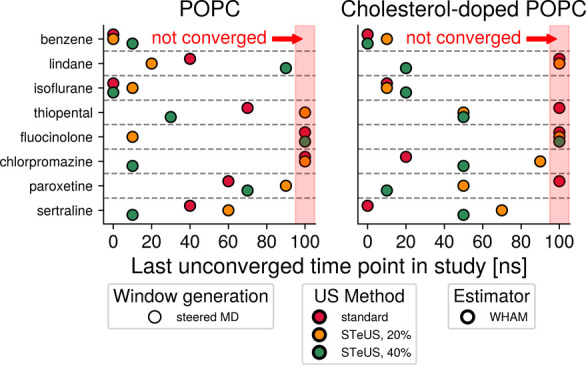
Influence of the varying of the simulation method in isolation on convergence speeds. The effectiveness of STeUS (orange, green) compared to standard umbrella sampling (red) was tested on sMD-based windows (circles) and WHAM-calculated (black outline) PMFs. Two different ground-state occupancies—20 and 40%—were tested. The results of the convergence speed analysis described in Figure 4 are shown here and demonstrate that STeUS can indeed be a powerful accelerant of convergence speeds, provided that an appropriate choice of ground-state occupancy is made. Markers placed at 100 ns represent umbrella sampling workflows that did not lead to convergence.

### Temperature Reweighting with MBAR Is Highly Effective in Small Systems

One downside of using simulated tempering for umbrella sampling is the reduction of simulation time that is spent sampling the temperature of interest. STeUS addresses this problem by allowing the specification of the desired relative ground-state occupancy. However, in its original publication with ground-state occupancies of 20 and 50% tested, this still means that 50 to 80% of simulation data is not used to inform the potential of mean force.

Here, we found that temperature reweighting can be a considerable accelerant of convergence. The PMFs of STeUS simulations carried out on sMD-based windows were calculated with MBAR, employing temperature reweighting to maximize data utilization for PMF calculation. Such calculated PMF profiles were found to converge as fast or faster than WHAM-calculated PMFs without temperature reweighting in 31 out of 32 WHAM-MBAR comparisons (Figure 7). Thus, the inclusion of data points sampled at the higher rungs on the STeUS temperature ladder demonstrably improves PMF convergence speeds. While the MBAR calculation requires more computational resources than WHAM, these additional requirements are more than made up by saving up to tens of nanoseconds of simulation time per umbrella window.

**Figure 7:**
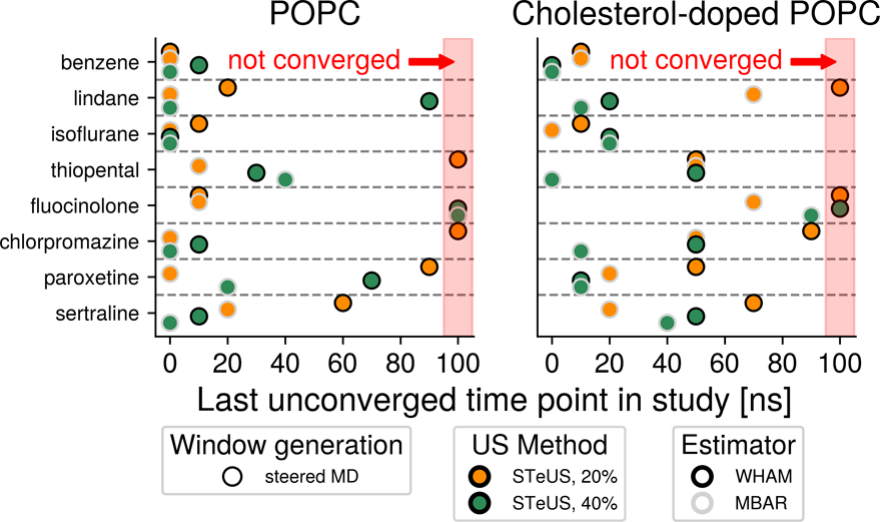
Effectiveness of temperature reweighting on increasing convergence speeds. STeUS simulations sample a temperature ladder, and traditionally, only data collected at the ground-state temperature is used to calculate the PMF as it is a temperature-dependent quantity (WHAM, black outline). However, in theory, higher-temperature data can be used to inform the ground-state PMF through temperature reweighting. Here, we compare convergence speeds with and without temperature reweighting, which is conveniently implemented in MBAR (gray outline). The effectiveness of temperature reweighting is reduced in larger systems (Figure S2F). However, we find that temperature reweighting is a highly useful accelerant of convergence in systems of this size (Table 1). Markers placed at 100 ns represent umbrella sampling workflows that did not lead to convergence.

The clear improvement is another compelling reason to reduce the size of the simulation system as much as possible, as the effectiveness of temperature reweighting reduces with increasing number of atoms^65^ (Figure S2F). Smaller systems are thus both faster to simulate and faster to converge, thanks to more meaningful temperature reweighting.

### Appropriate Choice of Tools Facilitates Calculation of Converged PMFs

While Figures 5, 6, and 7 demonstrate the effect of nonstandard components individually, Figure 8 shows convergence speeds for all combinations of the window generation method, umbrella sampling parameters, and statistical estimators tested in this study. This reveals synergistic effects on convergence speeds, in addition to the individual improvements of each component.

**Figure 8:**
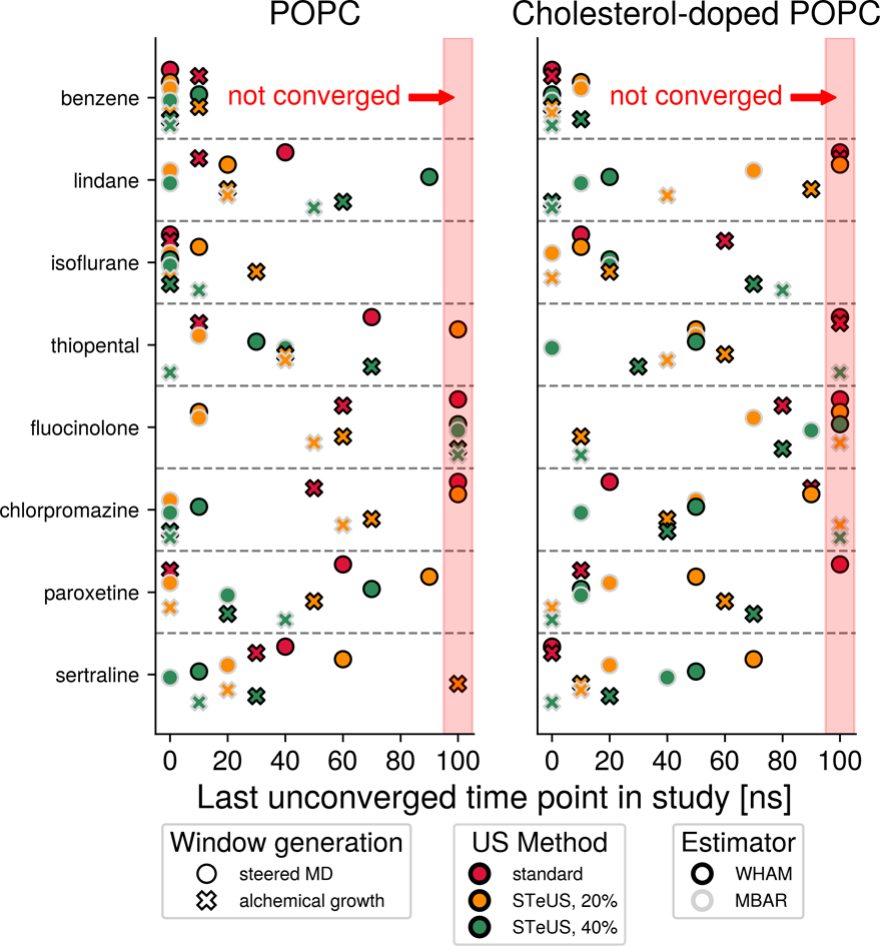
Overall comparison of convergence speeds of all combinations of window generation (shape), umbrella sampling method (fill color), and statistical estimator (outline color). This figure expands on the data shown in Figures 5, 6, and 7, which only compared the impact of varying tools for one of the three workflow steps while using the most commonly applied tools (sMD-based windows, standard umbrella sampling, and WHAM without temperature reweighting) for the other two steps. Here, the synergies of various combinations of tools are visible. Crucially, for all compounds but the easily converged benzene, an inappropriate choice of workflow parameters can delay convergence by tens of nanoseconds, while good choices of tools allow each system considered here to reach convergence within at most 20 ns per window. Markers placed at 100 ns represent umbrella sampling workflows that did not lead to convergence. An alternative representation of this data is included in Figure S7.

Figures 8 and 9 (and Figure S7) reveals that each compound-bilayer combination tested in the study has at least one set of workflow components that resulted in PMF convergence within at most 20 ns of simulation time per umbrella window. Unfortunate choices of components can prevent convergence even with 100 ns of simulation time per window. Individually, alchemically grown starting configurations, STeUS, and temperature reweighting with MBAR perform better than sMD-based windows, standard umbrella sampling, and WHAM on ground-state data only, respectively. It is mildly surprising, therefore, that the combination of these three “improved” components is not always the best workflow. In fact, workflows consisting of alchemical window growth, STeUS, and MBAR are among the best performers in 11 of 16 compound-bilayer combinations. In the case of lindane, fluocinolone, and sertraline in the POPC bilayer and thiopental and chlorpromazine in the cholesterol-doped POPC bilayer, other combinations of workflow components perform better. This appears to be caused by the combination of alchemical growth and STeUS underperforming slightly, while temperature reweighting with MBAR remains powerful in all comparisons.

**Figure 9:**
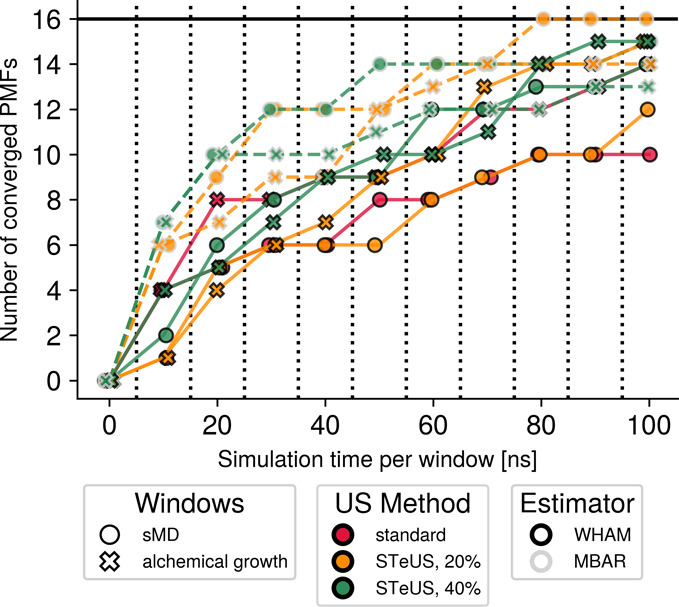
Alternative representation of the overall comparison of the effectiveness of each tested umbrella sampling workflow. The number of converged compound-bilayer combinations at each simulation end point is shown. For the eight compounds and two lipid bilayers tested here, a total of 16 combinations are available. The method that led to the largest number of converged conditions after 100 ns per window is STeUS with 20% ground-state occupancy and temperature reweighting run on sMD-based windows. However, other workflows may lead to faster convergence depending on the compound-bilayer combination. The 10 ns end point illustrates this well, where PMFs of 7 out of 16 systems are already converged for STeUS with 40% ground-state occupancy, instead. In general, alchemically grown windows tend to outperform sMD-based windows, STeUS tends to outperform standard umbrella sampling, and temperature reweighting with MBAR tends to improve PMFs.

In general, PMF convergence in the cholesterol-doped POPC bilayer appears to be slower. Considering the higher complexity of this bilayer, this is to be expected.

### Well-Converged PMFs Show Features Consistent with Experiments

Through the appropriate choice of window generation method, umbrella sampling parameters, and statistical estimator in the umbrella sampling workflow, well-converged PMF profiles can be obtained with drastically reduced computational effort. Example profiles for each compound and bilayer are shown in Figure 10 (solid lines and Figure S3). Symmetrical and periodic PMF profiles were obtained for each compound-bilayer combination. Comparison of profiles obtained for the two bilayers shows that umbrella sampling is sensitive enough to show both the thickening of cholesterol-containing bilayers and the reduced partitioning of these eight compounds with increased cholesterol content. Both of these observations thus agree with experimental evidence.^24,27^

**Figure 10:**
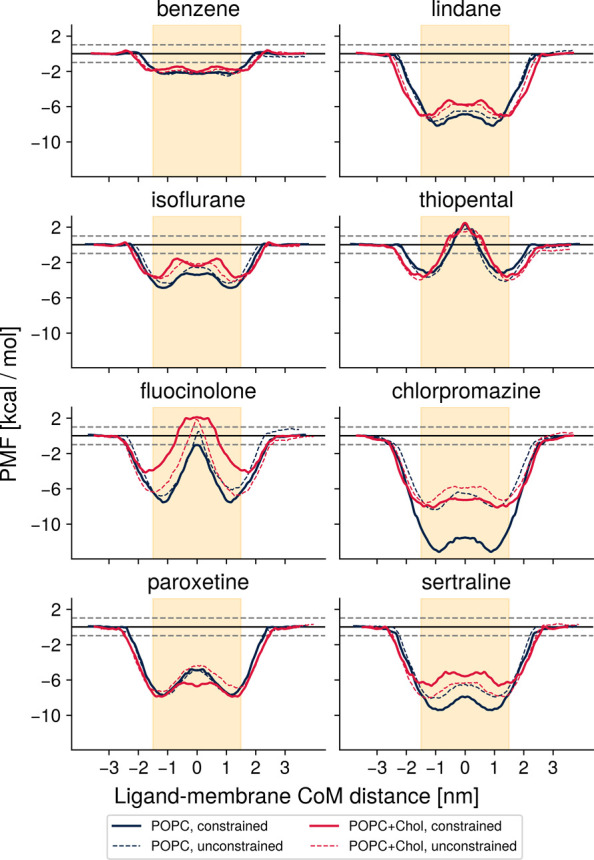
Comparison of converged, unconstrained PMF profiles (dashed lines) and profiles calculated with constraints of periodicity and symmetry (solid lines) for all solute-bilayer combinations. Chemical accuracy (±1 kcal/mol) is shown with dashed gray lines. The orange shading indicates the hydrophobic core of the bilayer. The partition coefficient into this core was determined (Figure 11). Umbrella sampling is sufficiently sensitive to detect the cholesterol-induced widening of the lipid bilayer. Constrained and unconstrained PMFs are very similar for easy-to-converge ligands such as benzene, but stark differences are apparent for more complicated compounds such as chlorpromazine or fluocinolone acetonide. The profile of any converged condition (see Figure 8) could have been chosen for display here as they have converged to nearly identical results (Figure S4). The profiles displayed here were chosen because they corresponded to the fastest-converging conditions for each ligand (Table S2).

### Enforcing Symmetry and Periodicity of PMF Profiles Leads to Errors

Commonly, only very brief simulations of each window are carried out in umbrella sampling studies. This often leads to insufficient sampling and errors in the PMF calculation. Some WHAM implementations allow the application of boundary conditions, such as symmetry or periodicity of the free-energy surface, to make the PMF profile better match the simulation system.

In Figure 10, we compare our well-converged PMF profiles to those calculated with such boundary conditions to determine what, if any, error arises from their application. Our aim was to illuminate what error is to be expected in a typical umbrella sampling study that makes use of these constraints when insufficient sampling was carried out. To that end, we calculated PMF profiles based on 10 ns of simulation time on sMD-based windows under the standard umbrella sampling protocol with gmx wham, enforcing symmetry and periodicity through use of the relevant flags, as profiles for this condition and end point were for the most part not yet converged (Figure 8)

Unsurprisingly, the differences in PMF profiles are smallest for easy-to-converge compounds. As benzene and isoflurane, for example, are already close to convergence at 10 ns, the differences between profiles calculated with and without constraints are minor. More complicated systems such as those with fluocinolone or chlorpromazine, on the other hand, show drastic differences between PMF profiles. This illustrates that the systems are still far from convergence after 10 ns of simulation time, as can be seen in the asymmetry of the purple PMF profile in Figure 4B for example. Enforcing symmetry and periodicity of the PMF profile at this stage introduces large errors along the entire length of the profile. The profiles are made to look convincing but are far from converged, correct results. The underlying sampling issues that led to PMF asymmetry are obscured when these constraints are applied.

To quantify these errors, we used the PMF profiles to calculate partition coefficients (Figure 11 and Table 4) of the compounds between the hydrophobic core of the bilayer (orange shading in Figure 10) and the aqueous bulk solvent. We carried out the calculation for all combinations of workflow components that led to converged PMFs in this study (Figure 8 and Figure S4). These partition coefficients were compared to those calculated from constrained PMF profiles, which were obtained from three independent sMD-based standard umbrella sampling repeats with only 10 ns per window (Figure S5). The comparison shows that well-converged systems reliably identify a statistically significant reduction of partitioning into cholesterol-doped POPC bilayers. This is no longer ensured in the PMFs calculated with the external constraints, with only the reduction of isoflurane partitioning reaching statistical significance. The errors associated with the application of boundary conditions enforcing symmetry and periodicity are particularly apparent in the case of fluocinolone, chlorpromazine, and sertraline. Experimental values do not provide a perfect comparison, as a variety of lipid compositions, cholesterol concentrations, and temperatures were used in the literature. Furthermore, as we were interested in convergence speeds rather than comparison with experiment, we performed no optimization of ligand parameters. With these caveats in mind, for the compounds tested here, reductions of log*K*_p_ of 0.1–0.6 are reported (Table S1). In this study, differences in log*K*_p_ values calculated from converged PMFs reliably fell within this range, while coefficients calculated from constrained profiles for fluocinolone, chlorpromazine, and sertraline were well outside it.

**Figure 11:**
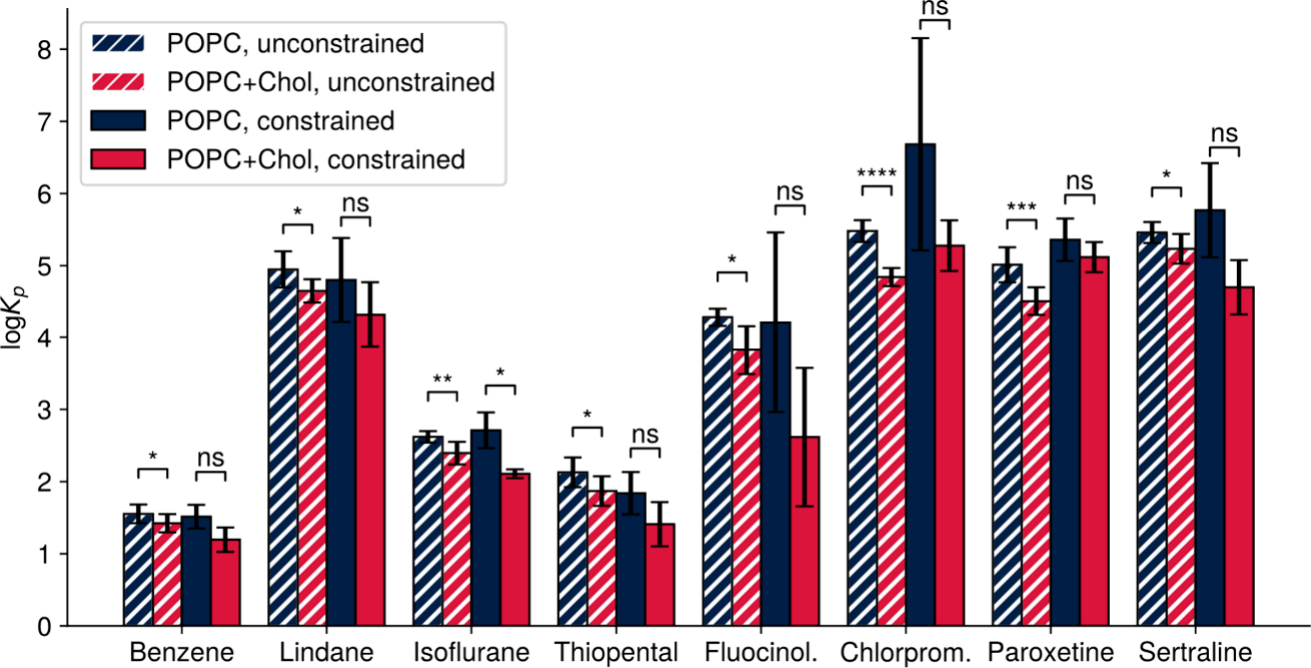
log*K*_p_ values calculated from PMF profiles. Partition coefficients between bulk solvent and the hydrophobic core (orange shading in Figure 10) were calculated from the PMF profiles. Shown are the mean ± standard deviation of all converged conditions (unconstrained calculations) or of three independent repeats of constrained PMF calculations (10 ns of standard umbrella sampling on sMD-based windows, PMF calculated with WHAM with periodicity and symmetry constraints). Tabulated data is available in Table 4. Statistical significance was tested with the two-sided Student’s *t*-test implementation ttest_ind available in SciPy.^66^ log*K*_p_ values calculated from unconstrained, converged profiles reliably identify a statistically significant reduction of ligand partitioning into cholesterol-containing bilayers. This is not the case for PMFs calculated with WHAM constraints, where only the reduction of isoflurane partitioning is statistically significant. ns: not significant. *: *p* < 0.05. **: *p* < 0.01. ***: *p* < 0.001. ****: *p* < 0.0001. Solid and hatched bars are used to match the solid and dashed lines in Figure 10.

**Table 4:** Calculated log*K*_p_ Values^a^

	**calculated log*K*_p_ (mean** ± **standard deviation)**			
compound	POPC	POPC+Chol	POPC, constraints	POPC+Chol, constraints
**benzene**	1.553 ± 0.129 (*n* = 10)	1.423 ± 0.127 (*n* = 10)	1.514 ± 0.164 (*n* = 3)	1.195 ± 0.169 (*n* = 3)
**lindane**	4.948 ± 0.251 (*n* = 10)	4.651 ± 0.162 (*n* = 7)	4.801 ± 0.582 (*n* = 3)	4.32 ± 0.451 (*n* = 3)
**isoflurane**	2.619 ± 0.078 (*n* = 10)	2.395 ± 0.156 (*n* = 10)	2.71 ± 0.248 (*n* = 3)	2.108 ± 0.061 (*n* = 3)
**thiopental**	2.128 ± 0.206 (*n* = 9)	1.869 ± 0.205 (*n* = 7)	1.838 ± 0.294 (*n* = 3)	1.41 ± 0.307 (*n* = 3)
**fluocinolone acetonide**	4.286 ± 0.119 (*n* = 5)	3.825 ± 0.337 (*n* = 6)	4.212 ± 1.249 (*n* = 3)	2.617 ± 0.96 (*n* = 3)
**chlorpromazine**	5.481 ± 0.15 (*n* = 8)	4.843 ± 0.124 (*n* = 8)	6.681 ± 1.47 (*n* = 3)	5.278 ± 0.351 (*n* = 3)
**paroxetine**	5.013 ± 0.243 (*n* = 10)	4.509 ± 0.191 (*n* = 9)	5.36 ± 0.293 (*n* = 3)	5.119 ± 0.209 (*n* = 3)
**sertraline**	5.461 ± 0.146 (*n* = 9)	5.235 ± 0.205 (*n* = 10)	5.768 ± 0.653 (*n* = 3)	4.7 ± 0.376 (*n* = 3)

a Listed are mean log*K*_p_ values and standard deviations. In the case of unconstrained PMF calculations, only combinations of window generation method, simulation parameters, and statistical estimator that yielded converged PMFs in this study were used for log*K*_p_ calculation (*n* ≤ 10 profiles, confer Fig 8). These properly converged PMFs are juxtaposed with those obtained with commonly used workflows that involve WHAM constraints of symmetry and periodicity. We ran three independent repeats of standard umbrella sampling on sMD-based windows with 10 ns of simulation per window and calculated the PMF with gmx wham with the -cycl, -sym, and -ac flags set.

## Discussion

In this work, we set out to test the performance of various membrane permeation umbrella sampling strategies to come up with recommendations for best practices. We chose to simulate the permeation of eight different small molecules (Figure 1) through two different lipid bilayers (Figure 2). In the first part of this work, we tested 10 combinations of window generation methods (alchemical growth of system configurations or seeding from steered MD), umbrella sampling parameters (standard umbrella sampling or STeUS^21^ with varying ground-state occupancy), and statistical estimation (WHAM or MBAR with temperature reweighting on STeUS simulations) and determined how much simulation time is required to obtain converged PMF profiles for each combination. This gave us 10 repeats for each compound-bilayer system and required the collection of ∼730 μs of simulation data. We defined convergence through two error metrics of symmetry (Figure 4) as we simulated the bilayer systems under periodic boundary conditions and thus expected near perfect symmetry of sufficiently sampled PMFs. This convergence speed analysis gave numerous insights (Figure 8). Benzene was expected to display rapid convergence, as it is a very small and rigid molecule. Indeed, we observed that the choice of umbrella sampling workflow barely matters in its case, as all tested combinations lead to converged PMFs in less than 20 ns of simulation time per window. The bulkier, more complicated ligands tested here are more sensitive to the choice of workflow. Importantly, we observed that some compound-bilayer combinations did not yield converged PMFs within 100 ns in some workflows but converged rapidly with different sets of workflow components. This is particularly evident in the cases of thiopental, fluocinolone acetonide, and chlorpromazine (Figure 8). Our results stress the importance of making appropriate choices in the umbrella sampling workflow.

However, we are unable to recommend a single combination of window generation methods, umbrella sampling parameters, and statistical estimator as the single best workflow. While alchemical growth of configurations, STeUS, and temperature reweighting with MBAR all perform better than their more commonly used counterparts (Figures 5, 6 and 7), their combination was not always the best-performing workflow. This appears to be caused by slight underperformance of the combination of alchemical growth and STeUS, while temperature reweighting remains powerful regardless of the window generation method. We believe this might be explained by the fact that in our alchemically grown windows, the relative starting orientations of the ligand are identical. Extracting windows from a steered-MD trajectory, on the other hand, introduces inherent variability in solute poses between windows (Figure S6). It may be that this variability helps accelerate the convergence of bulkier ligands such as fluocinolone. To test this hypothesis further, additional studies should be undertaken in which the initial solute orientation in alchemically grown windows is randomized. This heterogeneity would match windows obtained from steered MD more closely and thus eliminate this possible source of reduced performance.

Nevertheless, a clear result of this study is that umbrella sampling workflows should be carefully assembled to maximize convergence speeds. Our recommendation is that at the outset of new umbrella sampling studies, convergence speed trials should be run to make a deliberate choice of workflow components. Umbrella windows should be obtained with different methods, and short simulations (perhaps 1–5 ns) with different simulation methods should be run. Comparison of PMF profiles calculated from these short simulations can then give clues as to which method is likely to lead to convergence the fastest. While these initial trials are work-intensive, they make up for it by saving up to tens of nanoseconds of simulation time per window, which corresponds to savings of microseconds of simulation time overall.

We have thus demonstrated that converged PMFs of membrane permeations are computationally accessible, even in the case of chemically complicated ligands and multicomponent bilayers with modern hardware. On older hardware, such simulation times were much less readily accessible and indeed windows of around 1 ns were practical choices combined with a correction to enforce the expected symmetry.^15,18^ However, this approach has the inherent risk that PMFs that are far from convergence will be made to look converged by design. This caution was previously raised by Markthaler et al., who reported minor differences in estimated standard binding free enthalpies between such constrained and unconstrained calculations, and recommended the elimination of underlying sampling issues instead.^19^

Here, we set out to examine the magnitude of errors that can be introduced into unconverged PMF profiles by enforcing their symmetry and periodicity with WHAM in a standard umbrella sampling workflow on steered-MD based windows. To this end, we created three sets of windows for each compound-bilayer system from independent sMD trajectories, ran standard umbrella sampling for 10 ns per window, and calculated PMFs with gmx wham supplied with the -cycl, -sym, and -ac flags. The resulting PMFs for easily converged compounds such as benzene and isoflurane are very similar to each other (Figure S5) and to their unconstrained, properly converged counterparts (Figure 10). This makes intuitive sense—if the profile is already close to being converged, enforced symmetry and periodicity will reduce noise without meaningfully changing the profile otherwise. Compounds like fluocinolone acetonide, chlorpromazine, or sertraline, on the other hand, are much slower to converge and are far from convergence at the 10 ns time point. The use of the WHAM constraints at this point leads to profiles that vary strongly between each other and from the unconstrained, converged PMFs. This constitutes the first caution against using these constraints as a crutch to make PMFs look better.

To further quantify these effects, we calculated the partition coefficients (log*K*_p_) between the hydrophobic membrane core and the bulk solvent of each ligand. The average log*K*_p_s for all converged umbrella sampling workflows (between *n* = 5 and *n* = 10, see Table 4) were compared to those obtained for the constrained PMFs calculated in triplicate (Figure 11) There is experimental evidence for all eight compounds tested in this study that their partitioning into membranes is reduced at increased cholesterol concentrations, with log*K*_p_ reductions ranging roughly from 0.1 to 0.6 (Table S1). Significance testing with Student’s *t*-test revealed that only log*K*_p_ values obtained from the converged, unconstrained PMF profiles allow reliable identification of this reduction in partitioning. Calculations based on constrained profiles, on the other hand, reach statistical significance only in the case of thiopental. log*K*_p_ values of fluocinolone acetonide, chlorpromazine, and sertraline appear particularly problematic due to their stark differences between bilayers and the large standard deviations. Calculating constrained PMFs based on insufficient data can thus lead to faulty conclusions.

We have thus provided evidence that the errors introduced by PMF symmetrization are not necessarily negligible and can give results that are magnitudes removed from those of converged experiments. We therefore argue that their use should be avoided. Instead, calculation of properly converged PMF profiles is now accessible through hardware improvements and the availability of new enhanced sampling methods constantly under development,^21,67–72^ avoiding this source of error.

## Conclusions

This work provides important benchmarking of common techniques in umbrella sampling and makes recommendations for future studies to help accelerate PMF convergence. We have demonstrated that variation of the tools used in the umbrella sampling workflow can have a huge impact on convergence speeds and recommend that a screening of different techniques be carried out at the outset of future permeation studies to choose the optimal conditions for the system at hand and save computational resources. Beyond the detailed benchmarking and comparison of established methods, the key contributions of this study are the following. We demonstrated the problems with steered-MD-based window generation clearly and provided evidence that alchemical growth is a superior window generation method. This approach has previously been used, for example by Wennberg et al.,^15^ but the present study is to our knowledge the first direct comparison of the impacts of convergence speed of either method. Furthermore, we found that Simulated Tempering-enhanced Umbrella Sampling^21^—a powerful accelerant of convergence in itself—can strongly benefit from temperature reweighting if the simulation system is small enough. Finally, we also provide a description of the magnitude of errors that can be introduced by enforcing symmetry and periodicity on unconverged PMF profiles. We thus caution against the use of such constraints in all cases except for the denoising of already converged PMFs.

## Supplementary Material



## Notes

Literature values for log*K*_p_ reductions due to cholesterol, details on the choice of PMF profiles displayed in Figure 10, quantification of cholesterol flip-flop and evaluation of restraint effectiveness, further details on the use of temperature reweighting with MBAR, additional PMF profiles, and alternative representation of the convergence speed information shown in Figure 8 (PDF)
