# Supplementary material for: Umbrella Sampling Workflows for Fast-Converging PMF Calculations without Artificial WHAM Constraints
Source: J Chem Theory Comput. 2026 May 6;22(10):5299–310. doi: 10.1021/acs.jctc.6c00246 (PMC13217548; doi:10.1021/acs.jctc.6c00246)
Supplement: Supplementary file 1 [file ct6c00246_si_001.pdf]

Supporting Information

Umbrella sampling workflows for fast-converging PMF calculations without artificial WHAM constraints

Bjarne Feddersen and Philip C. Biggin*

Structural Bioinformatics and Computational Biochemistry, Department of Biochemistry, University of Oxford, South Parks Road, Oxford, OX1 3QU,

UK

*E-mail: philip.biggin@bioch.ox.ac.uk

SI Table S1. Literature values for the reduction of solute partitioning into cholesterol-containing lipid bilayers. These values are very approximate and reported for a variety of different lipid compositions, temperatures, and final cholesterol concentrations. As our goal in this study was not to match experimental data but instead to determine PMF convergence speeds, we did not aim to replicate either experimental conditions nor results. The values reported here are merely intended to provide context for the analysis shown in Fig. 11 to show what reductions in $\log K_p$ are reasonable. Roughly, reductions in $\log K_p$ between 0.1 and 0.6 were reported, with values around 0.5 most commonly seen.

Compound	K_p (without Chol)	K_p (with Chol)	Reduction in $\log K_p$	Reference
Benzene	~4000	~1000	3.6 → 3	De Young et al[1]
Lindane	~2000	~700	3.3 → 2.8	Antunes-Madeira et al[2]
Isoflurane	165	49	2.2 → 1.7	Dickinson *et al* [3]
Thiopental	437	126	2.6 → 2.1	Korten *et al* [4]
Fluocinolone	48500	~17000	4.7 → 4.2	Takegami *et al* [5]
Chlorpromazine	5400	2300	3.7 → 3.4	Luxnat *et al* [6]
Paroxetine	146000	113000	5.16 → 5.05	Ngo *et al*[7]
Sertraline	276000	208000	5.44 → 5.32	Ngo *et al*[7]

SI Table S2. Workflow parameters underlying the unconstrained PMF profiles shown in Fig. 10 of main manuscript and SI Fig. S3. List of window generation method, umbrella sampling protocol, and statistical estimator of the PMF profiles. These particular profiles are shown because they correspond to the fastest-converging combinations for each ligand. As all converged profiles have converged to the same result, any of them could have been chosen for visualisation (SI Fig. S4).

Compound	Pure POPC bilayer	Cholesterol-doped POPC bilayer
**Benzene**	Alch. growth, STeUS (40 %), MBAR	Alch. growth, STeUS (40 %), MBAR
**Lindane**	sMD, STeUS (40 %), MBAR	Alch. growth, STeUS (40 %), MBAR
**Isoflurane**	Alch. growth, STeUS (40 %), WHAM	sMD, STeUS (20 %), MBAR
**Thiopental**	Alch. growth, STeUS (40 %), MBAR	sMD, STeUS (40 %), MBAR
**Fluocinolone**	sMD, STeUS (20 %), MBAR	Alch. growth, STeUS (40 %), MBAR
**Chlorpromazine**	Alch. growth, STeUS (40 %), MBAR	sMD, STeUS (40 %), MBAR
**Paroxetine**	sMD, STeUS (20 %), MBAR	Alch. growth, STeUS (20 %), MBAR
**Sertraline**	sMD, STeUS (40 %), MBAR	Alch. growth, STeUS (40 %), MBAR

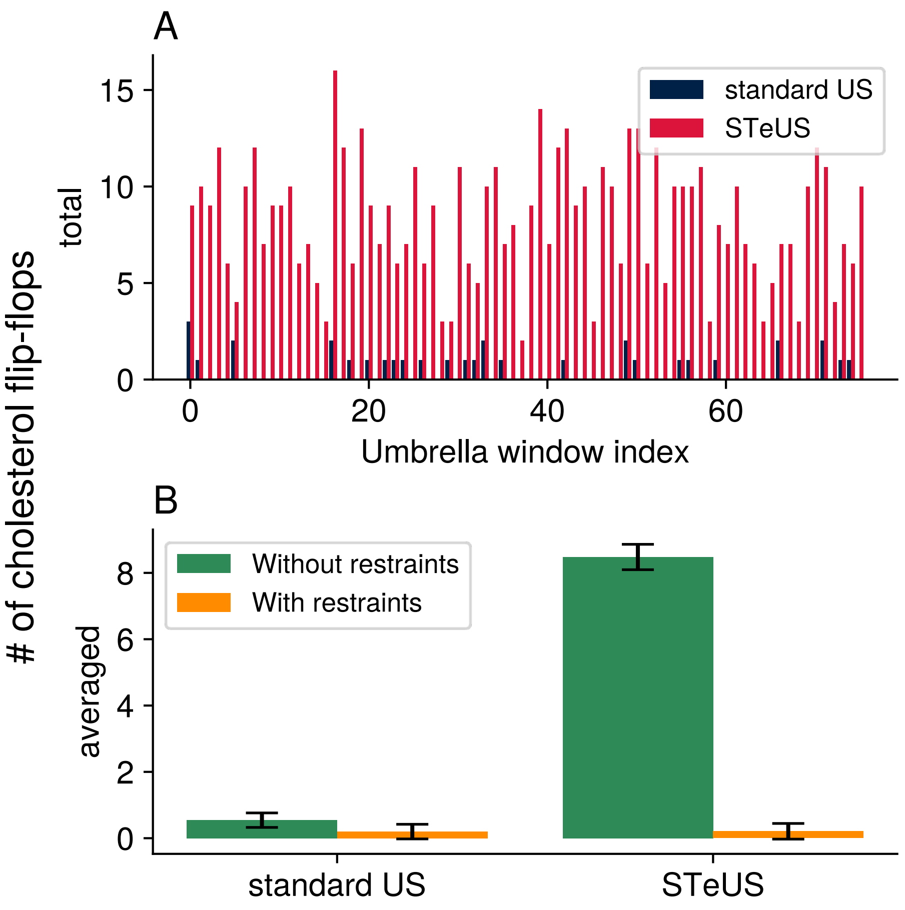

SI Figure S1. Cholesterol flip-flop observed in this study. A: Total number of cholesterol flip-flops observed in each of 76 windows of an example umbrella sampling system. Few flip-flops occur with standard umbrella sampling at 310 K. The increased temperature sampled in STeUS systems coincides with a stark increase of flip-flop events across all windows. Crucially, the number of flip-flops varies widely. Thus, the leaflet composition varies both within and between umbrella windows, which is problematic for the PMF calculation with WHAM. To avoid these problems in WHAM, harmonic, flat-bottom restraints are applied to the cholesterol head groups. These still allow free movement within leaflets while preventing moves between leaflets. B: Average flip-flop numbers across all systems show the effectiveness of these restraints at preventing flip-flopping.

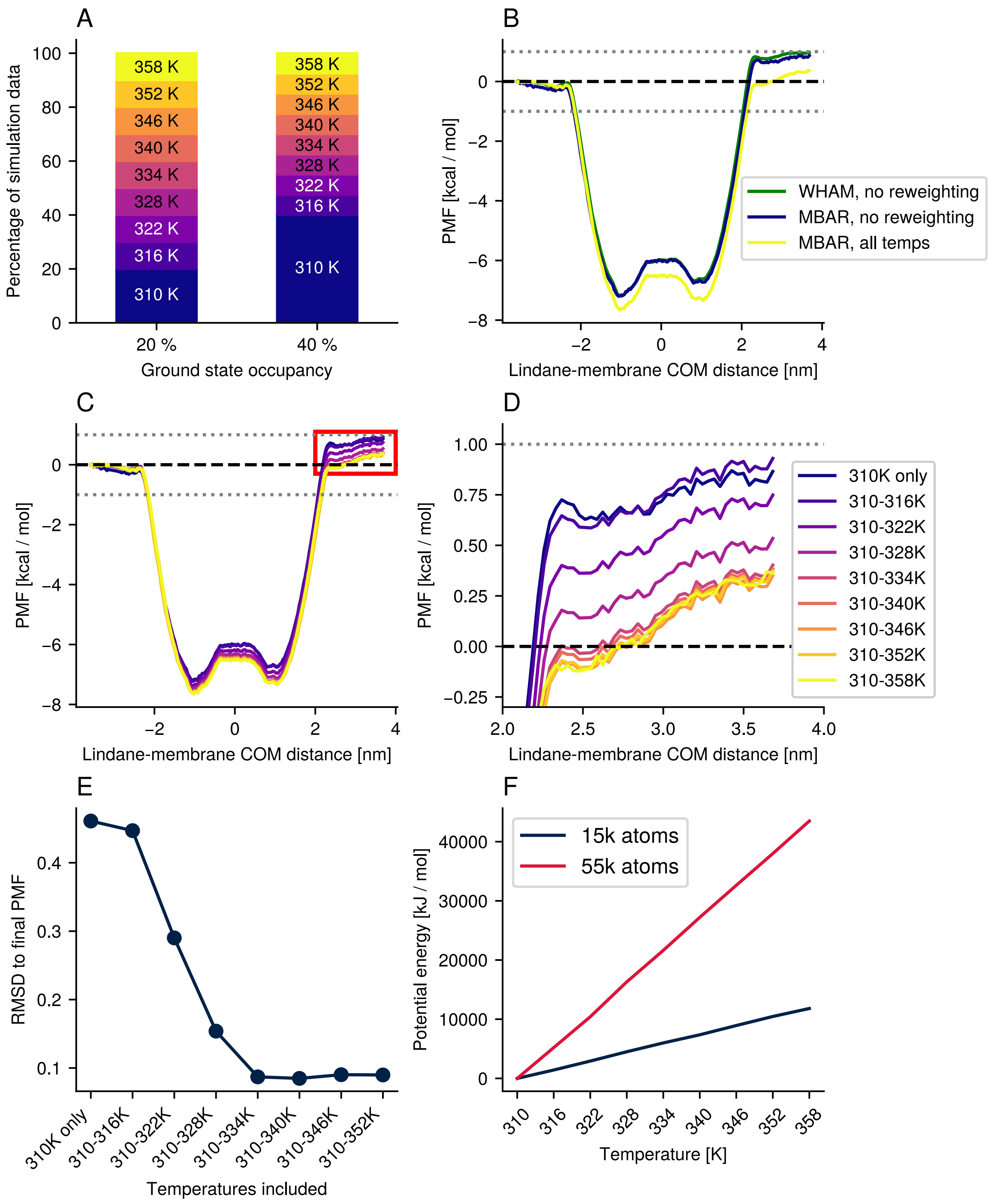

SI Figure S2. Impact of temperature reweighting with MBAR on PMF convergence. A: Illustration of the fraction of simulation time spent at each temperature. B: Comparison of the final PMFs for lindane on sMD-generated windows in the POPC bilayer, calculated from STeUS simulations with 40 % ground state occupancy. The PMF calculated with MBAR using temperature reweighting (yellow line) is markedly more symmetric than that calculated with WHAM (green line) which only took data collected at the ground state (310 K) into consideration. Running MBAR on ground state data only (blue line) gives a PMF very similar to that calculated with WHAM as expected. C: Impact of including data collected at higher temperatures in the PMF calculation. Inclusion of temperature steps closer to the ground state has a more profound impact on the PMF. Temperatures further removed barely affect the PMF calculation, as their relative weight is near zero. D: Close-up of the region highlighted in red in panel C. The diminishing returns of including data

beyond 334 K is clearly visible. E: RMSD values of PMF profiles a subset of temperature steps included, relative to the PMF calculated from the full 310–358 K range. The diminishing returns of including data from temperature steps beyond 334 K is again visualised. F: Effect of the system size (in number of atoms) on the change of the potential energy with the temperature. This is the key reason why temperature reweighting only works well in small systems. In larger systems, the gradient of the potential energy with respect to the temperature is steeper, which causes the relative weights to approach zero more quickly. The larger the system, and the larger the steps on the temperature ladder, the less information can be gained from temperature reweighting.

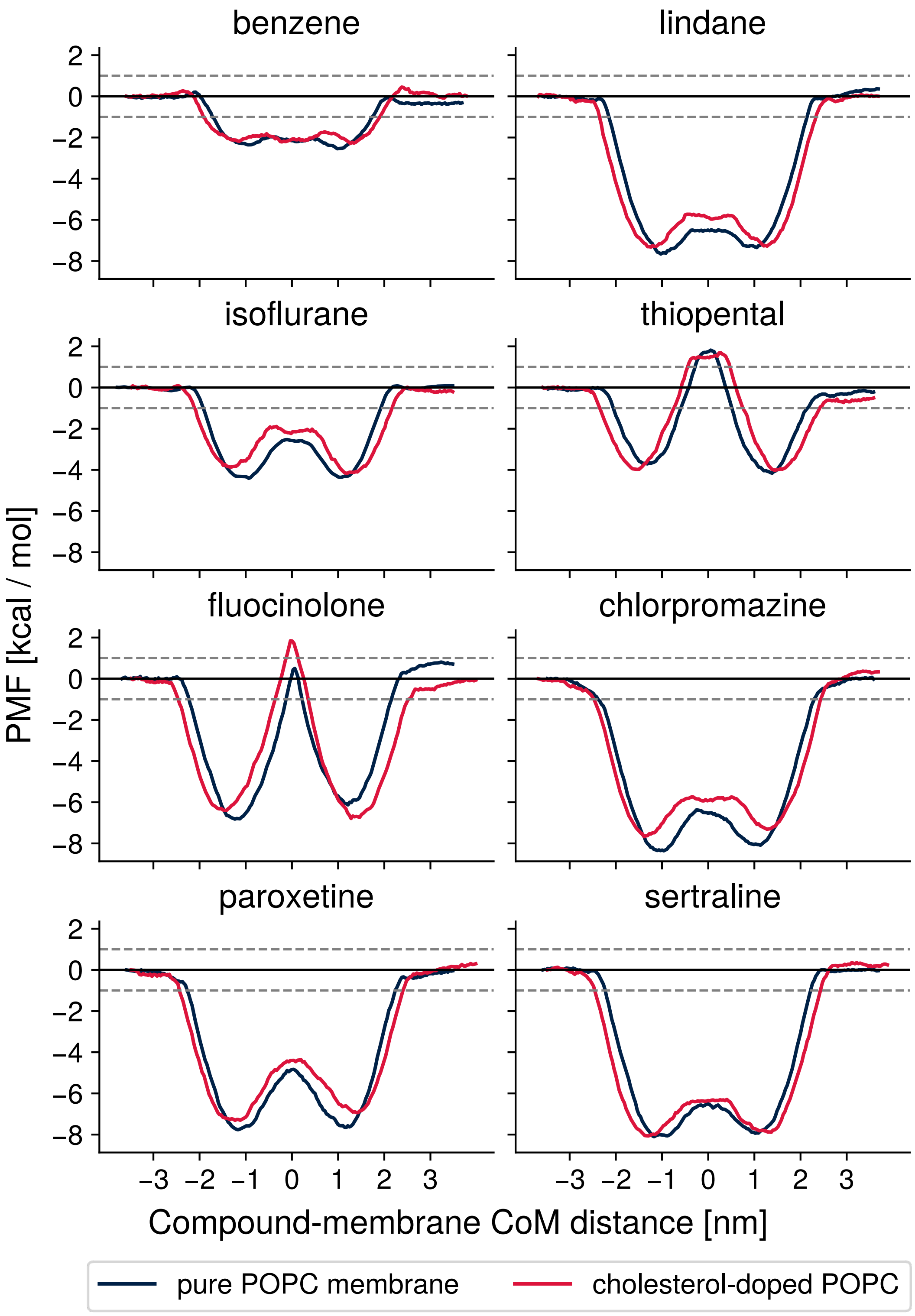

SI Figure S3. Examples of well-converged PMF profiles obtained in this study. Replicated from Fig. 10 of the main manuscript, shown here without the constrained PMF profiles for visual clarity. The underlying method of each profile is the fastest converging method for the system at hand (SI Table S2).

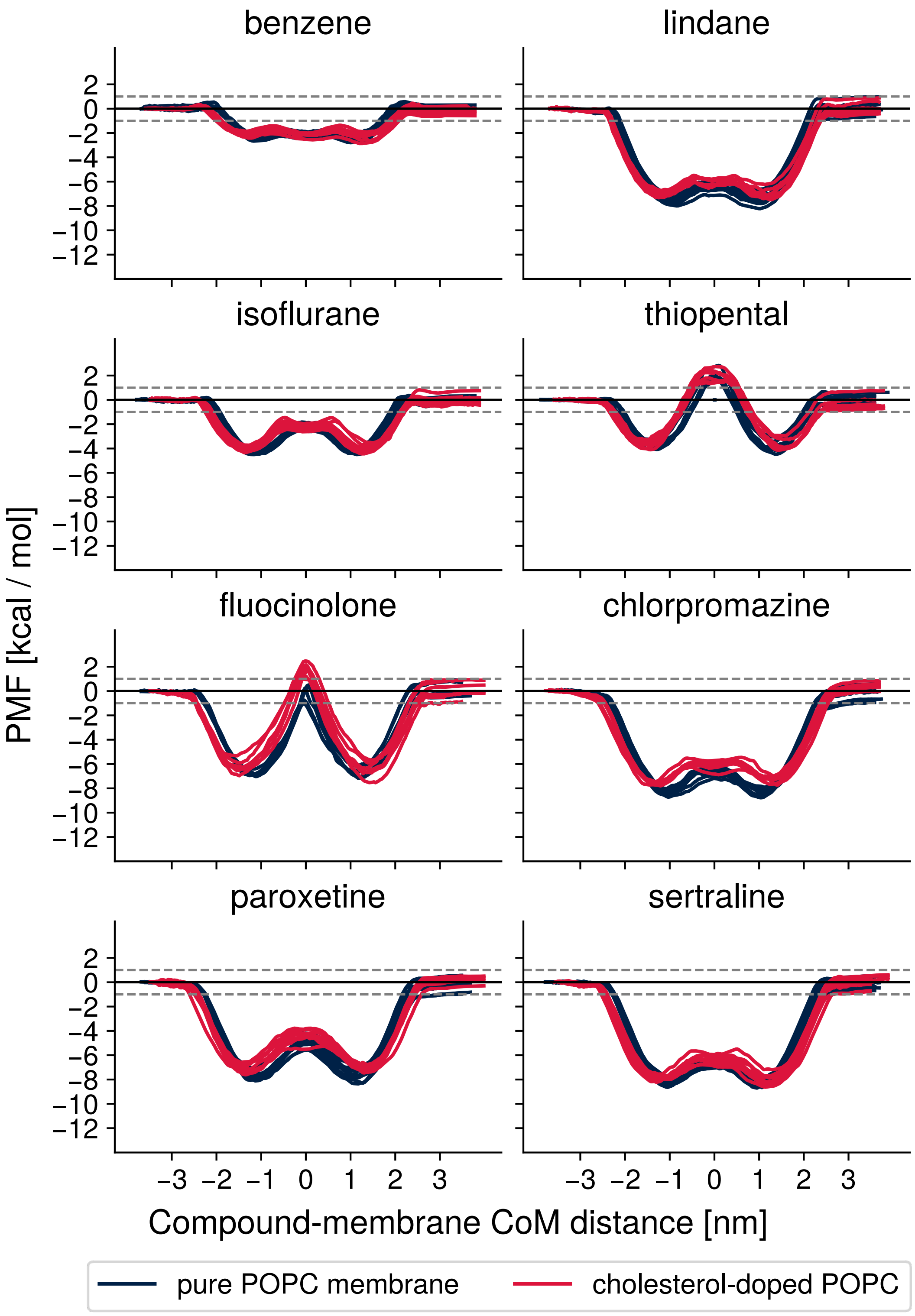

SI Figure S4. Overlay of all converged PMF profiles obtained for each ligand-bilayer combination. The profiles are highly similar, indicating that the choice of convergence metric outlined in Fig. 4 of the main manuscript is suitable and that any of them could have been chosen for display in Fig. 10 of main manuscript and SI Fig S3.

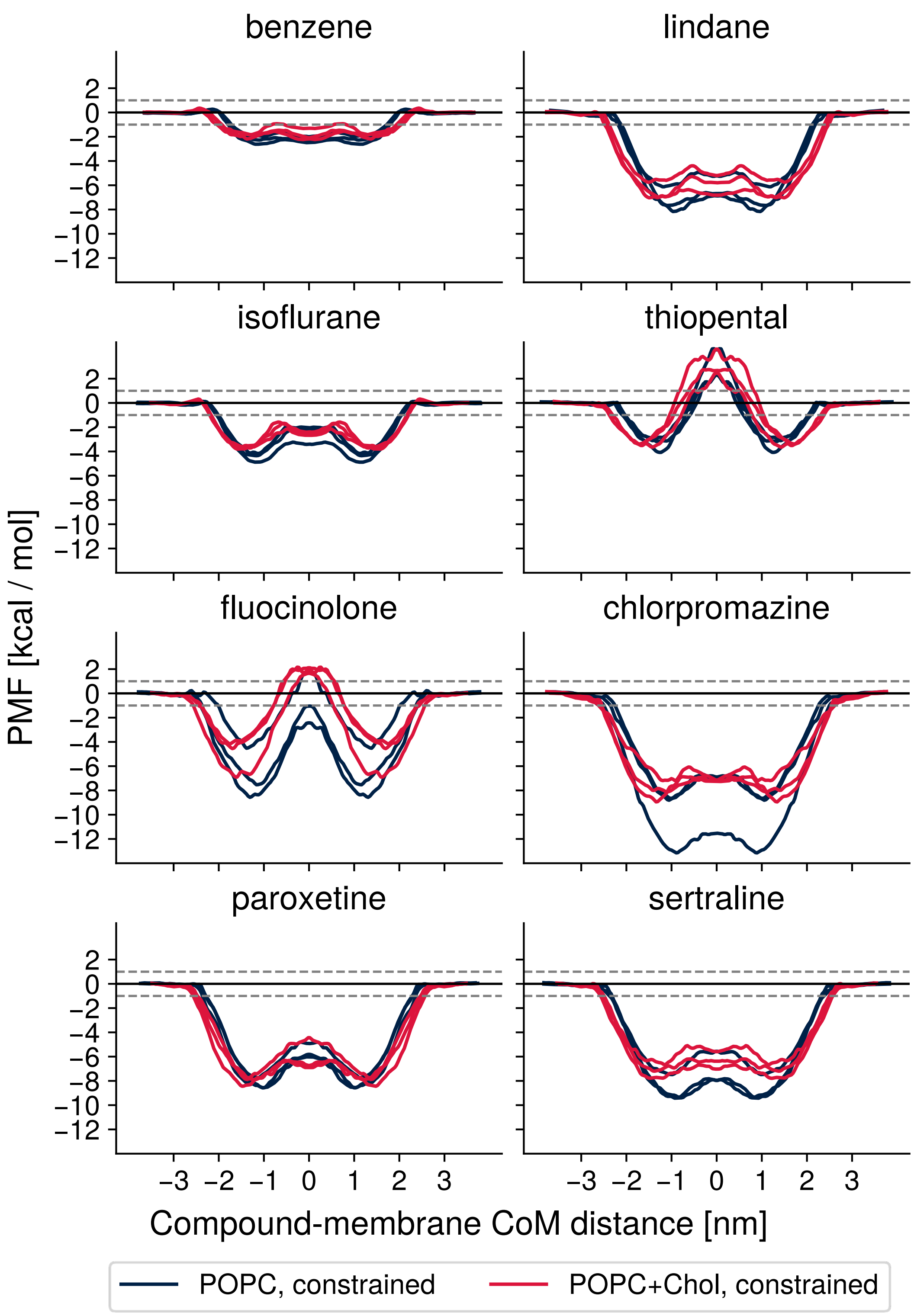

SI Figure S5. Overlay of the PMF profiles obtained with WHAM from data collected from sMD-based windows in 10 ns of standard umbrella sampling simulations. Periodicity and symmetry were enforced in the PMF calculation with the appropriate flags passed to gmx wham. The constraints yield profiles that meet the convergence criteria defined in Fig. 4 by design. However, the large degree of variance between profiles of the same system reveals lack of convergence.

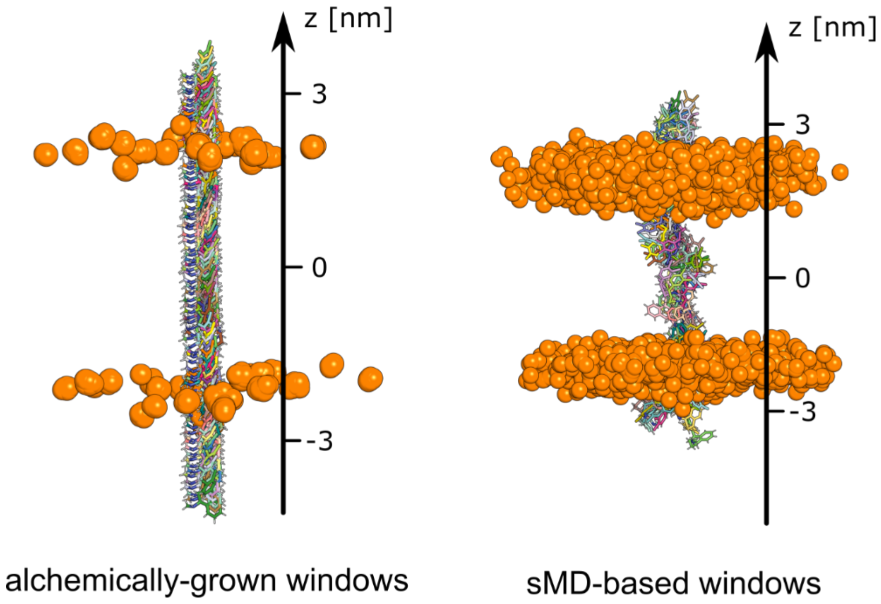

SI Figure S62. Comparison of alchemically-grown and sMD-based windows generated in this study. In the example shown, all 76 windows of sertraline in the POPC bilayer are overlayed. Wider fluctuations of the membrane are visible in set of sMD-based windows, which is a contributor to hysteresis. Alchemically-grown windows show much less variation of the simulation system between windows. However, as discussed in the main text, all alchemically-grown windows start with the same relative ligand orientation, while sMD-based windows have increased variability.

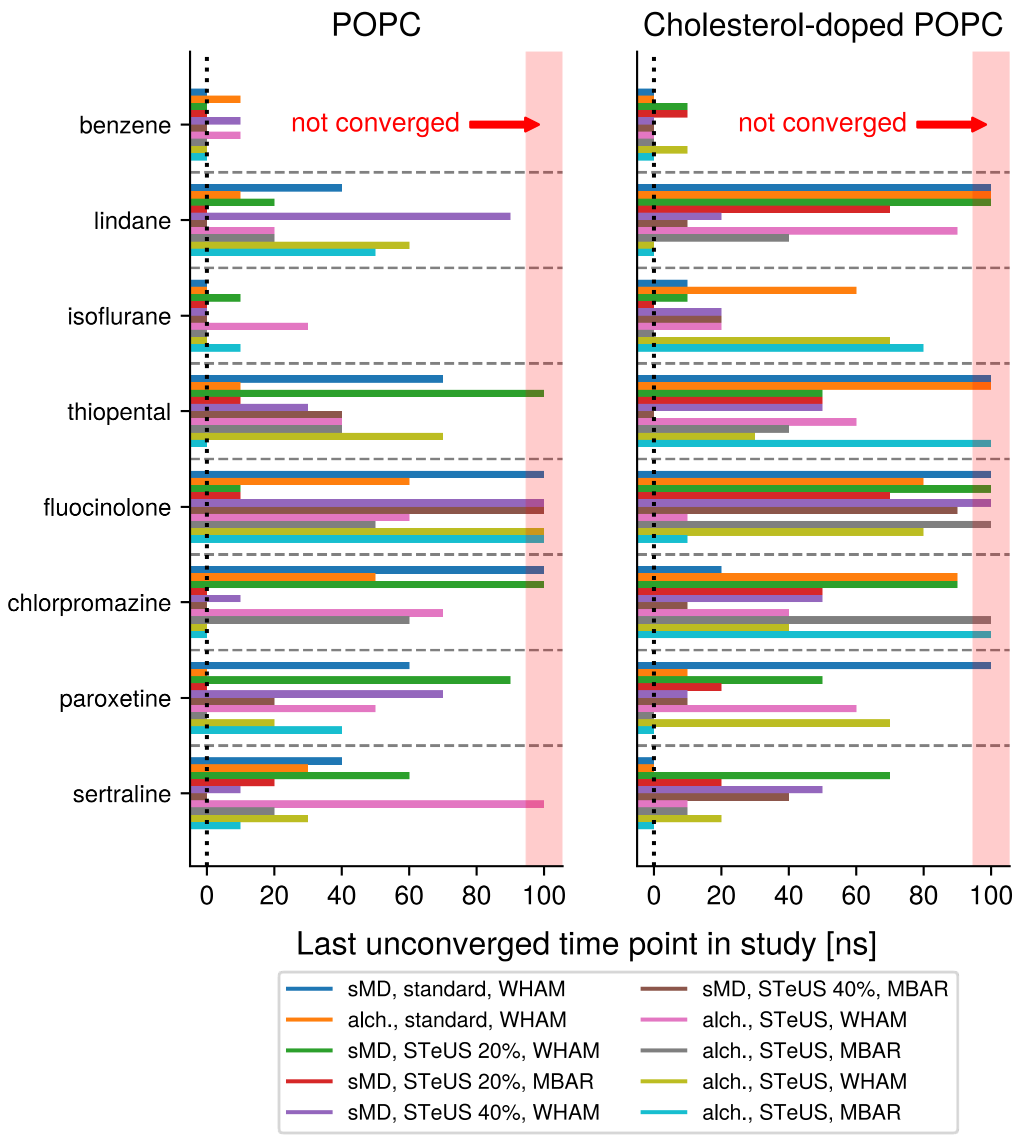

SI Figure S7: Alternative representation of the convergence speed data. Instead of distinguishing by marker shape for window generation, fill colour for simulation method, and edge colour for statistical estimator, the umbrella sampling workflow combinations are here identified via colour only. This representation reduces the risk of misidentifying a workflow but makes targeted comparisons between individual workflow components more challenging.

References

1. De Young, L. R. & Dill, K. A. Solute partitioning into lipid bilayer membranes. *Biochemistry* **27**, 5281–5289 (1988).
2. Antunes-Madeira, M. C. & Madeira, V. M. C. Partition of lindane in synthetic and native membranes. *Biochim. Biophys. Acta BBA - Biomembr.* **820**, 165–172 (1985).
3. Dickinson, R., Franks, N. P. & Lieb, W. R. Can the stereoselective effects of the anesthetic isoflurane be accounted for by lipid solubility? *Biophys. J.* **66**, 2019–2023 (1994).
4. Korten, K., Sommer, T. J. & Miller, K. W. Membrane composition modulates thiopental partitioning in bilayers and biomembranes. *Biochim. Biophys. Acta BBA - Biomembr.* **599**, 271–279 (1980).
5. Takegami, S., Kitamura, K., Funakoshi, T. & Kitade, T. Partitioning of anti-inflammatory steroid drugs into phosphatidylcholine and phosphatidylcholine-cholesterol small unilamellar vesicles as studied by second-derivative spectrophotometry. *Chem. Pharm. Bull. (Tokyo)* **56**, 663–667 (2008).
6. Luxnat, M. & Galla, H.-J. Partition of chlorpromazine into lipid bilayer membranes: the effect of membrane structure and composition. *Biochim. Biophys. Acta BBA - Biomembr.* **856**, 274–282 (1986).
7. Ngo, D. T. N., Ho, T. H., Huynh, L. K. & Nguyen, T. T. The interplay of membrane fluidity, acyl chain order and area per lipid on the partitioning of two antidepressants paroxetine and sertraline. *Soft Matter* **19**, 5527–5537 (2023).
